# Immunity and reproduction protective effects of Chitosan Oligosaccharides in Cyclophosphamide/Busulfan-induced premature ovarian failure model mice

**DOI:** 10.3389/fimmu.2023.1185921

**Published:** 2023-05-09

**Authors:** Xiaoyan Li, Haifeng Ye, Tie Su, Chuan Hu, Yaoqi Huang, Xinxin Fu, Zhisheng Zhong, Xuelian Du, Yuehui Zheng

**Affiliations:** ^1^ Biobank center, The Second Affiliated Hospital of Nanchang University, Nanchang, China; ^2^ Reproductive Health Department, Shenzhen Traditional Chinese Medicine Hospital, Shenzhen, China; ^3^ Department of Microbiology, Graduate School of Medical Sciences, Kumamoto University, Kumamoto, Japan; ^4^ Institute of Regenerative Biology and Medicine (IRBM), Helmholtz Zentrum München, Munich, Germany; ^5^ Department of Pathology, Yingtan People’s Hospital, Yingtan, China; ^6^ School of Basic Medicine, Nanchang University, Nanchang, China; ^7^ Reproductive Center of Obstetrics and Gynecology, The Second Affiliated Hospital of Nanchang University, Nanchang, China; ^8^ National Demonstration Center for Clinical Teaching & Training, Xiang’an Hospital of Xiamen University, Xiamen, China

**Keywords:** chitosan oligosaccharides, premature ovarian failure, ovarian function, immunological function, prophylaxis, therapy

## Abstract

**Introduction:**

Premature ovarian failure (POF) is a major cause of infertility among women of reproductive age. Unfortunately, there is no effective treatment available currently. Researchers have shown that immune disorders play a significant role in the development of POF. Moreover, growing evidence suggest that Chitosan Oligosaccharides (COS), which act as critical immunomodulators, may have a key role in preventing and treating a range of immune related reproductive diseases.

**Methods:**

KM mice (6-8 weeks) received a single intraperitoneal injection of cyclophosphamide (CY, 120mg/kg) and busulfan (BUS, 30mg/kg) to establish POF model. After completing the COS pre-treatment or post-treatment procedures, peritoneal resident macrophages (PRMs) were collected for neutral erythrophagocytosis assay to detect phagocytic activity. The thymus, spleen and ovary tissues were collected and weighed to calculate the organ indexes. Hematoxylin-eosin (HE) staining was performed to observe the histopathologic structure of those organs. The serum levels of estrogen (E2) and progesterone (P) were measured *via* the enzyme-linked immunosorbent assay (ELISA). The expression levels of immune factors including interleukin 2 (IL-2), interleukin 4 (IL-4), and tumor necrosis factor α (TNF-α), as well as germ cell markers Mouse Vasa Homologue (MVH) and Fragilis in ovarian tissue, were analyzed by Western blotting and qRT-PCR. In addition, ovarian cell senescence *via* p53/p21/p16 signaling was also detected.

**Results:**

The phagocytic function of PRMs and the structural integrity of thymus and spleen were preserved by COS treatment. The levels of certain immune factors in the ovaries of CY/BUS- induced POF mice were found to be altered, manifested as IL-2 and TNF-α experiencing a significant decline, and IL-4 presenting a notable increase. Both pre-treatment and post-treatment with COS were shown to be protective effects against the damage to ovarian structure caused by CY/BUS. Senescence-associated β-galactosidase (SA-β-Gal) staining results showed that COS prevents CY/BUS-induced ovarian cell senescence. Additionally, COS regulated estrogen and progesterone levels, enhanced follicular development, and blocked ovarian cellular p53/p21/p16 signaling which participating in cell senescence.

**Conclusion:**

COS is a potent preventative and therapeutic medicine for premature ovarian failure by enhancing both the ovarian local and systemic immune response as well as inhibiting germ cell senescence.

## 1 Introduction

Premature ovarian failure (POF), also known as premature ovarian insufficiency (POI), is becoming increasing prevalent recently (1, 2). This condition is characterized by a significant impairment in ovarian function, which has a serious impact on a woman’s physical health and overall well-being. POF not only affects the production of eggs and sex hormones, but also contributes to female infertility (3). Approximately 1-5% of women under the age of 40 experiencing infertility due to POF. As the reproductive system ages 20-30 years earlier than other systems in the body, the effects of reproductive aging can have a profound impact on a woman’s daily life (4, 5). Given the significant increase in life expectancy over the past century, the gap between menopause and life expectancy is growing larger, making it increasingly important to effectively prevent and treat POF.

Chitosan oligosaccharide (COS) is a biopolymer that is widely recognized for its valuable immunopotentiating properties (6). Laboratory studies have shown that chitosan and its derivatives have immunomodulatory and anti-inflammatory effects with minimal toxicity and mild side effects (7–9). Additionally, COS has been found to have protective effects against hydrogen peroxide-induced damage by functioning as an antioxidant and attenuating the production of reactive oxygen species (ROS), malondialdehyde (MDA), and lactate dehydrogenase (LDH) (10). Furthermore, COS inhibits angiotensin converting enzyme (ACE) and prolyl endopeptidase (PEP) by binding specifically to the active site of enzymes and competing with their natural substrate (11, 12). Studies have also shown that dietary of chitosan can inhibit hypercholesterolemia and atherogenesis in the apolipoprotein E-deficient mouse model of atherosclerosis. Moreover, COS has shown therapeutic effects in treating various neuronal disorders, including Alzheimer’s disease and Parkinson’s disease (13). In 2020, it was reported that the concentrations of spw serum immunoglobulin (IgA, IgG, IgM) and secretory immunoglobulin A (sIgA), the success rate of pregnancy and serum IgM and sIgA concentrations of piglets were significantly increased by regular dietary of COS supplementation. It shows that COS has a significant effect on the reproductive system (14).

From a biogenetic and evolutionary viewpoint, the immune system can be categorized into non-specific (innate) and specific (adaptive) immunity, with the latter further branching into humoral immunity and cell-mediated immunity (15, 16). Although the immune system operates relatively independently from other systems, it plays a vital role in comprehensively maintaining an individual’s homeostasis (17). Some studieshave shown a correlation between the immune system and ovarian function, particularly through their synchronous interactions during the aging process (18–20). The immune system regulates reproductive function throughout an individual’s life, from the perinatal stage to post-menopause (21). In humans, the thymus is the organ that first undergoes senescence, followed by the ovary. Lintern-Moore and colleaguesfound that follicle loss in athymic nude mice was evident at an early age of two months due to a reduction in the number of primordial and medium follicles in the initial growth stages. They also noted a delay in the first ovulation until 2.5 months of age (22). Likewise, a study showed ovarian hypoplasia in neonatal mice after thymectomy could be restored by administering thymocytes from normal mice. Furthermore, the addition of exogenous thymulin to athymic mice which were performed thymectomy on 10-day-olds of age resulted in an earlier onset of puberty, with a decrease in the weights of ovaries and uterus. However, a combination of pregnant mare serum gonadotrophin(PMSG) and human chorionic gonadotrophin (hCG) as well as thymulin was able to restore the ovulation onset time as well as the weights of ovaries and uterus (23). These findings suggest that the presence of the thymus after the neonatal period is necessary for normal ovarian development and function. Furthermore, accumulating studies have confirmed that immune cells and cytokines can regulate the secretion of sex hormones in the ovary (24, 25).

In our earlier researches, we investigated how COS can safeguard granulosa cells from harm and enhance the function of ovarian germ stem cells by modulating the HIF-1α and SESN2/NRF2 signaling pathway (26–28). As the well-being of follicular cells is closely linked to the system and ovarian local immune status, it is crucial to explore the *in vivo* ovarian protective role and mechanism of COS possessingpotential as a treatment for POF.

In this study, we utilized the cyclophosphamide/busulfan-induced POF model to investigate the potential preventive or therapeutic effects of COS on POF-induced ovarian function damage. We aimed to investigate whether COS achieves this effect by regulating the immune system to alleviate ovarian senescence.

## 2 Materials and methods

### 2.1 Mice

Female mice of Kunming strain, aged 6-8 weeks, were procured purchased from the Center of Experimental Animals at Nanchang University. The mice were housed under standardized conditions, with a constant temperature of 22 ± 2°C,humidity between 40-60%, and a 12-hour light/dark schedule. They were free access to laboratory chow and water. All trials were conducted in compliance with the Regulations of the People’s Republic of China on the Administration of Laboratory Animals. The Research Ethics Committee of Nanchang University provided their consent for the study (NO. NCULAE-20220928003).

### 2.2 Mice treatment protocol

**Figure 1:**
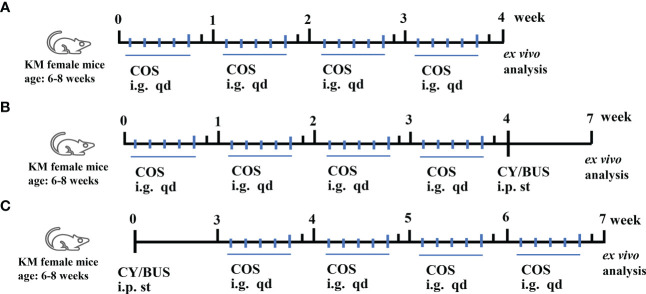
Mice treatment procedures. Mice received single, prophylactic or therapeutic applications of COS. **(A)** Mice were given COS (200mg/kg/d) intragastrically every day for the first five days of each week for four weeks, and ovarian and immune functions were assessed at the end of the experiment to evaluate any potential toxicity. **(B)** Mice received prophylactic treatment with varying doses of COS (100/200/300mg/kg/d) for five days each week, followed by a single intraperitoneal injection of CY/BUS. **(C)** Mice were given a single intraperitoneal injection of CY/BUS before undergoing a four-week therapeutic treatment with increasing concentrations of COS (100/200/300mg/kg/d). *Ex vivo* analyses were done as described.

The preparation of CY/BUS and COS (GlycoBio, China) was carried out according to previously published methods (27, 29). Briefly, cyclophosphamide and busulfan, obtained from Sigma (USA), were dissolved together in physiological saline solution or dimethyl sulfoxide at a concentration of 12mg/mL and 3mg/mL respectively. The mice were weighed and intraperitoneally injected cyclophosphamide (120mg/kg) and busulfan (30mg/kg) according to their body weight. **Figure 1** shows the treatment procedures used in this study. In COS treatment (200 mg/kg/d) (n=6) group, normal mice were administered the treatment intragastrically once a day for the first five days of each week for four weeks. In the COS prophylaxis groups (n=6 for each group), mice were given intragastric COS at concentrations of 100, 200 or 300 mg/kg/d for the first five days of each week for four weeks, followed by a single intraperitoneal injection of CY/BUS. In the COS therapy groups (n=6 for each group), mice were given a single intraperitoneal injection of CY/BUS and then underwent COS therapy at increasing concentrations (100/200/300 mg/kg/d) for four weeks. Mice with a single injection of CY/BUS were used as a control for premature ovarian failure (POF), n=6. Normal control mice (n=6) were given intraperitoneal injection of a mixture of normal saline and dimethyl sulfoxide, followed by free diet and regular environment feeding. At the experimental endpoint, ovarian and immune functions were evaluated as described below.

### 2.3 Serum, PRMs, spleen, thymus and ovary collection

Blood, peripheral blood mononuclear cells (PRMs), spleen, thymus, and ovary were collected in a sterile manner after measuring the body weight. The blood was coagulated at room temperature, and the serum was obtained through centrifugation. The tissues were washed with PBS, gently wiped to remove surface liquid, and then weighed and recorded. The organ/body weight index was calculated using the following formula:


Organ(ovary, spleen or thymus) index(‰)=organ(ovary, thymus or spleen)weight/body weight×1000

### 2.4 PRMs neutral red uptake assay

The PRMs neutral red uptake assay was conducted following previously described methods. Briefly, fetal bovine serum was administered intraperitoneally to stimulate PRM production three days before the experiment. On the day of the experiment, the mice were euthanized by neck broken after anesthesia, and a pre-cooled RPMI-1640 culture solution was injected into the abdominal cavity to stimulate PRMs precipitation. The abdominal fluid was collected and centrifuged to wash the PRMs. The washed PRMs were then plated onto a 96-well dish at a concentration of 1×10^6^ cells/ml and cultured in an incubator at 37°C with 5% CO_2_ for 4 hours. After un-adherent cells were washed away with PBS, 0.1% neutral red solution was added. The cells were cultured for an additional 20 minutes, then observed the phagocytosis of neutral red by PRMs under an Olympus IX71 microscope and photographed. After washed with PBS, cells were lysed with a solution of acetic acid and anhydrous ethanol in a 1:1 ratio. The absorbance was measured at 570nm after the cells were set at room temperature for 2 hours.

### 2.5 HE staining

After the collection of tissues, they were fixed overnight in 4% paraformaldehyde (PFA) and then embedded in paraffin blocks with a consistent embedding direction for each tissue type. Prior to embedding in paraffin, the tissues underwent dehydration using gradient alcohol and were made transparent using xylene. Next, the tissues were sequentially immersed in xylene, paraffinI, and paraffin II, each for a duration of 1 hour at 64 degrees Celsius. To perform hematoxylin-eosin (HE) staining, 5-µm sections were obtained and stained using the HE staining procedure (Baso, China). The maximum section in each tissue was selected for microscopic (Nikon Ci-S, Japan) observation on the pathological structure of tissue and photography, following previously established protocols (30).

### 2.6 Western blot analysis

The ovarian tissues were processed by cutting into small pieces and lysing with RIPA buffer (Beyotime, P0013C) using ultrasonic waves. The protein samples in SDS-OAGE loading buffer (G2075, Servicebio) were separated through electrophoresis on a 10% SDS-PAGE gel (Boster, USA) and transferred to a PVDF membrane (Millipore, USA). Primary antibodies, including anti-GAPDH (1:1000, ab181602, Abcam), anti-fragilis (1:200, ab18976, Abcam), anti-MVH (1:5000, ab27591, Abcam), anti-TNF-α (1:200, ab1793, Abcam), anti-IL-2 (1:200, ab11510, Abcam), anti-IL-4 (1:1000, 66142-1-Ig, Proteintech), anti-p16 (1:2000, ab51243, Abcam), anti-p21(1:1000, 28248-1-AP, Proteintech), and anti-p53 (1:5000, 60283-2-Ig, Proteintech), were incubated overnight after blocking with 5% skim milk for one hour. After washing with TBS-T, membranes were incubated with secondary antibodies (1:5000) for one hour at room temperature. All protein bands were imaged using the ChemiDoc XRS system (Bio-Rad, USA) and analyzed with ImageJ software. All experiments were repeated at least three times.

### 2.7 Quantitative real-time polymerase chain reaction

The TRIzol^®^ reagent (Qiagen, China) was utilized to extract total RNA from ovarian tissues, followed by processing of cDNAs using the PrimeScript RT kit and gDNA Eraser (TaKaRa, Japan). The expression levels were determined by the ABI7000 PCR instrument (Applied Biosystems, USA), with the primer sequences listed in **Supplementary Table 1**. GAPDH was utilized as a reference gene to normalize the expression results. Every sample was amplified in triplicate. The experiments were performed at least three times to ensure accuracy.

### 2.8 Serum estradiol and progesterone measurement

Blood samples were kept at room temperature for one hour before being centrifuged at 500g for 30 minutes. The resulting supernatant was collected and analyzed for levels of E2 and P using ELISA kits from Uscn Life Science (China) and Crystal Chem (USA), respectively. The manufacturer’s instructions were followed for the assays.

### 2.9 Senescence-associated β-galactosidase staining

SA-β-galactosidase staining was performed on mice ovaries using a Cellular Senescence Assay Kit (CBA-230; Cell Biolabs Inc. USA) according to the manufacturer’s protocol. In roughly, after fixation of the ovarian tissue overnight, it was thoroughly washed with PBS. The tissue was then incubated in a dyeing working solution overnight at 37°C. Following this, the tissue was washed again with PBS and subjected to the paraffin embedding process for embedding and sectioning. The resulting 5μm paraffin sections were dewaxed and sealed with neutral resin before being observed under a Nikon Ci-S (Japan) microscope and photographed. The location and color intensity of SA-β-galactosidase staining in tissues were observed.

### 2.10 Statistical methods

The data were presented as mean ± standard deviation (SD). GraphPad Prism 5.0 (GraphPad Software, Inc., CA) was used for statistical analysis. To test for differences between the Control group and COS group, Student’s t-tests were employed. On the other hand, one-way ANOVA tests were used to compare COS pre-treatment groups with Control and CY/BUS groups. In a similar vein, the one-way ANOVA tests were utilized to compare differences in COS post-treatment groups with Control group, and the CY/BUS group. Statistical significance was determined with a threshold of p<0.05, while p<0.01 and p<0.001 indicated high and very high significance, respectively.

## 3 Results

### 3.1 Protective and therapeutic effects of COS on systemic immune destruction in the CY/BUS-POF model

**Figure 2:**
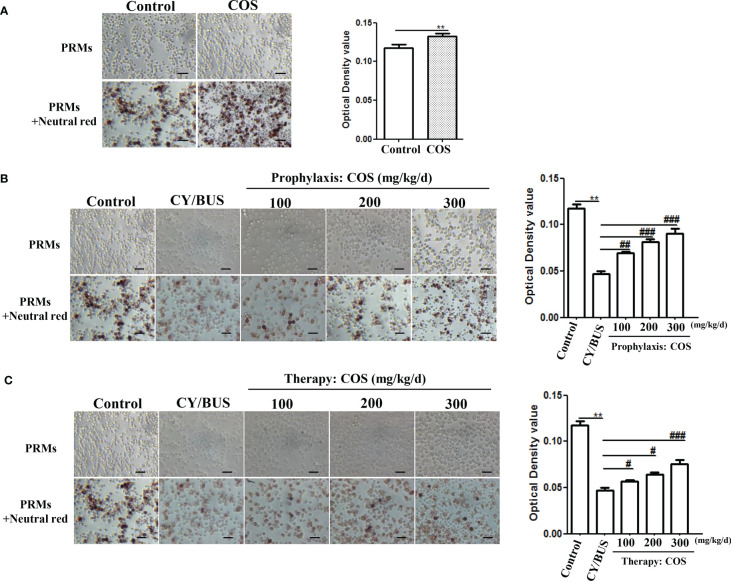
COS enhance PRMs phagocytosis ability analyzed by Neutral Red staining. **(A)** Mice PRMs were collected and phagocytosis capacity were determined by Neutral Red assay after 4 weeks COS (200 mg/kg. d) treatment or not. COS increasing the PRMs phagocytosis compared with normal controls. Left: PRMs microscope images, right: statistical contrast of optical density (OD) value (n = 6, Mean ± SEM). **(B)** PRMs Neutral Red staining were carried out at the endpoint of COS prophylaxis procedure. COS prevented CY/BUS induced PRMs phagocytosis damage in a dose-dependent manner. Left: PRMs microscope images, right: statistical contrast of optical density (OD) value (n = 6, Mean ± SEM). **(C)** PRMs Neutral Red assay were done at the endpoint of COS therapy procedure. CY/BUS treatment decrease PRMs phagocytosis. This trend was dose-dependent reversed after COS therapy. Left: PRMs microscope images, right: statistical contrast of optical density (OD) value (n = 6, Mean ± SEM). **p<0.01, vs Control group. #p<0.05, ##p<0.01, ###p<0.001, vs CY/BUS group. Scale bars = 50μm.

Here we first detected the PRMs activity *via* neutral red uptake assay (**Figure 2**). Compared with normal control, the PRMs phagocytic activity in COS-treated (200 mg/kg/d) mice was increased (**Figure 2A**), while in CY/BUS-POF mice (**Figure 2B**), it was decreased. In the prophylaxis procedure (**Figure 2B**), pretreat with different doses of COS limited the decrease in phagocytosis activity of PRMs with dose-dependent manner. The cell absorbance after 300 mg/kg/d COS pretreatment was almost double that of CY/BUS-POF. In the therapy groups (**Figure 2C**), different doses of COS post-treatment all increased the PRMs phagocytosis activity. Compared with the corresponding COS treatment dose of prophylactic groups, the PRMs phagocytosis activity in the therapy groups were decreased.

**Figure 3:**
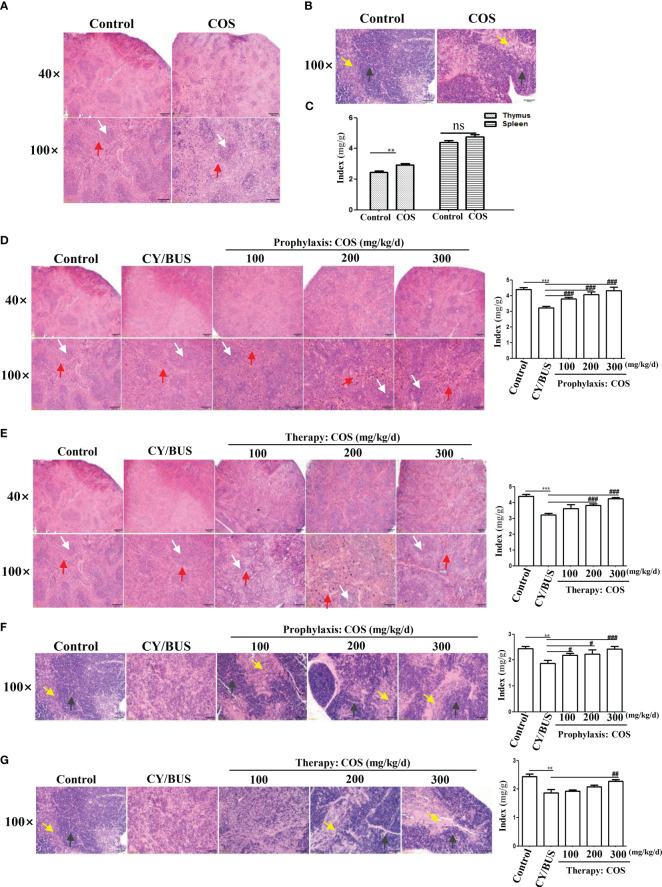
Size and histological changes of the spleen and thymus. Mice spleen and thymus were collected at each treatment endpoint then detected timely. The ratio index of spleen and thymus to body weight were used to evaluate the tissue size changes. Histological changes were examined by HE staining. **(A)** Spleen HE staining from mice with or without COS (200 mg/kg. d) treatment. **(B)** Thymus HE staining from mice with or without COS (200 mg/kg. d) treatment. **(C)** Changes of spleen and thymus index after COS (200 mg/kg. d) treatment. (**D, E**) At the endpoint of COS prophylaxis **(D)** or therapy **(E)** procedure, mice spleen were collected for HE staining (left) and index calculation (right). **(F, G)** After COS prophylaxis **(F)** or therapy **(G)** procedure, mice thymus were collected for HE staining (left) and index calculation (right). White arrows: white pulp; Red arrows: red pulp. Yellow arrows: medulla; Black arrows: cortex. n = 6, Mean ± SEM. ns: no significant difference. **p<0.01, ***p<0.001 vs Control group. #p<0.05, ##p<0.01, ###p<0.001, vs CY/BUS group. Scale bars = 200μm for 40X lens, Scale bars = 100μm for 100X lens.

We determined the pathological structure and organ index changes of the main immune organs, the thymus and spleen, to further analyze the protective effect of COS on systemic immunity in the CY/BUS-POF model (**Figure 3**). HE staining of the spleen (**Figure 3A**) and thymus (**Figure 3B**) revealed no significant difference in histological features between normal control mice and 200 mg/kg/d COS-treated mice. In the above two groups, the structure of red pulp and white pulp in spleen were recognizable with obvious boundary, uniform distribution. The pathological structure of thymus shows no obvious difference in single COS-treated mice compared to normal control mice (**Figure 3B**). COS (200 mg/kg/d) treatment had no effect on spleen index, but slightly increased thymus index (**Figure 3C**).

HE staining of spleen in CY/BUS-POF mice revealed lighter white pulp chromatin compared with normal control mice. The lighter staining center of the white pulp is the germinal center, which is rich in T-cells and B-cells. Germinal center was hard to observed in CY/BUS-POF mice spleen, and the spleen index was significantly decreased. After COS prophylactically (**Figure 3D**) or therapeutically treated (**Figure 3E**), the spleen index showed a dose-dependent increase, and the spleen pathological injury was improved compared with CY/BUS-POF mice. For thymus pathological structure and index changes, CY/BUS-POF mice thymus index was decreased and its pathological structure was disordered in thatit was difficult to distinguish cortex from medulla. Similarly, with COS prophylactic treatment (**Figure 3F**) and therapeutic treatment (**Figure 3G**), the thymus index showed a dose-dependent increase, and the pathological structure damage was improved as cortex and medulla clearly discernable.

### 3.2 COS improved CY/BUS-induced ovarian injury

**Figure 4:**
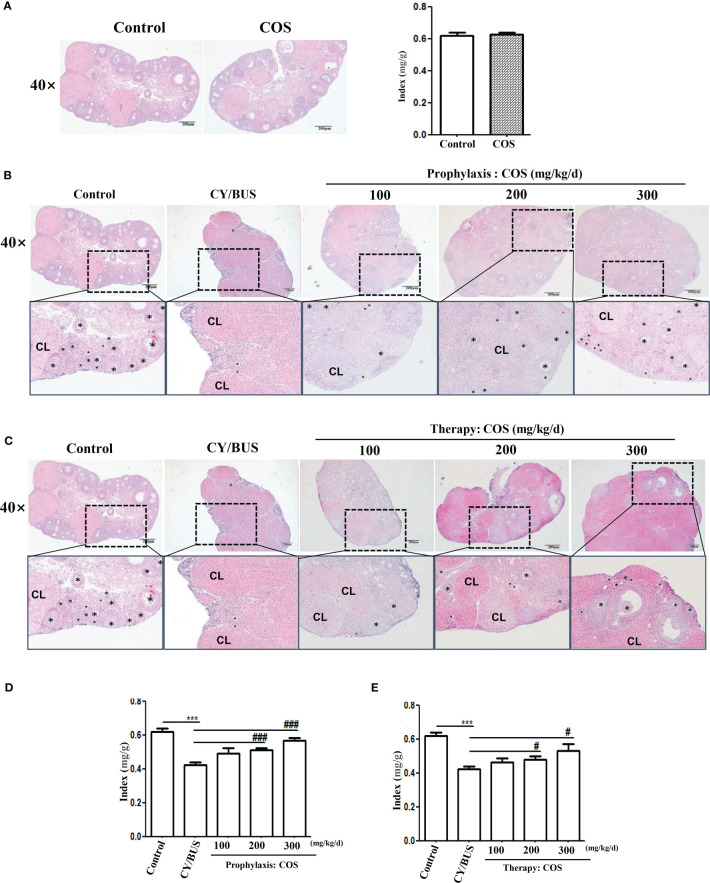
COS protect the CY/BUS induced morphological disruption of the ovary in a dose-dependent manner. At the end of each experimental procedure, the ovaries were removed and weighed to calculate the ovarian-to-body weight ratio. The ovaries were then sectioned and embedded in paraffin, and the maximum cross-section was stained with HE to examine the ovarian histopathological structure. **(A)** Ovary HE staining (left) and ratio index to body weight (right) from mice with or without COS (200 mg/kg. d) treatment. **(B)** Ovary HE staining from mice under prophylaxis treatment procedure. **(C)** Ovary HE staining from mice under therapy treatment procedure. **(D, E)** Mice ovary ratio index to body weight from prophylaxis procedure **(D)** and therapy procedure **(E)**. Black plum: follicle. CL: corpus luteum. n=6, Mean ± SEM. ns: no significant difference. ***p<0.001 vs Control group. #p<0.05, ###p<0.001, vs CY/BUS group. Scale bars = 200μm.

Follicular development and endocrinology were used to evaluate ovarian function. For ovarian function measurement, the ovarian index and histological evaluation *via* HE staining were conducted to assess ovarian development (**Figure 4**). The findings demonstrated that administration of COS (200 mg/kg/d) alone did not affect the ovarian index and pathological structure in normal mice (**Supplementary Table 2**, **Figure 4A**). However, pretreatment with various doses of COS prevented the reduction of ovarian index in a dose-dependent manner caused by CY/BUS, as shown in **Supplementary Table 3**, **Figure 4D**. Similarly, administering with COS after CY/BUS treatment improved the ovarian index in a dose-dependent manner (**Supplementary Table 4**, **Figure 4E**). COS exhibited beneficial effects on CY/BUS-induced retardation of follicle development. The results of HE staining of ovary sections with the largest cross-section showed that the total number of follicles was compared between each group through the microscope field with the largest number of follicles. It was observed that both COS pretreatment (**Figure 4B**) and post-treatment (**Figure 4C**) increased the follicles in CY/BUS-POF mice ovary.

**Figure 5:**
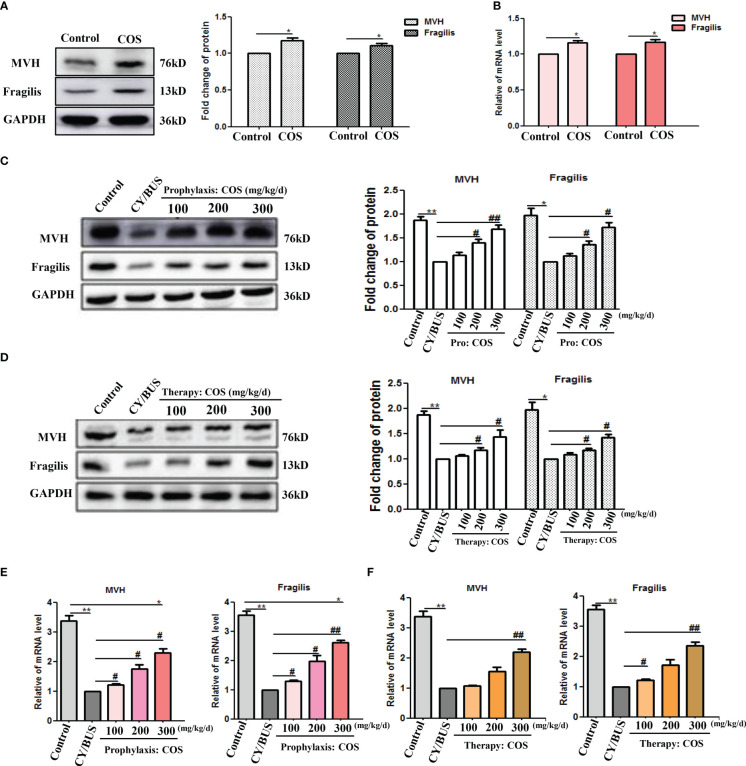
COS regulate mice ovarian germ-cell development. After drug treatment, total protein and mRNA were extracted from mouse ovaries and analyzed by western blot and qRT-PCR respectively to determine the development of germ cells. **(A, B)** MVH and Fragilis protein **(A)** and mRNA **(B)** lever in ovaries from mice treated with or without COS (200 mg/kg. d). **(C)** MVH and Fragilis protein detection results from mice ovaries after prophylaxis procedure. Left: western blot protein bands. Right: western blot densitometry values analysis result. **(D)** MVH and Fragilis protein detection results from mice ovaries after therapy procedure. Left: western blot protein bands. Right: western blot densitometry values analysis result. **(E, F)** MVH and Fragilis mRNA expression results in mice ovaries after prophylaxis procedure **(E)** and therapy procedure **(F)**. n=3, Mean ± SEM. *p<0.05, **p<0.01, ***p<0.001 vs Control group. #p<0.05, ##p<0.01 vs CY/BUS group.

To investigate ovary follicular development, we analyzed germ cell markers MVH and Fragilis (**Figure 5**, **Supplementary Figure 1**). Our Western blotting analysis demonstrated a slight increase in the protein and mRNA levels of both MVH and Fragilis in the ovaries of mice following direct administration of COS at a dose of 200 mg/kg/d (**Figures 5A, B**). The protein and mRNA levels of MVH and Fragilis were significantly reduced in the ovaries of CY/BUS-induced POF mice. While, COS pretreatment significantly inhibited the decline of both MVH and Fragilis protein (**Figure 5C**) and mRNA (**Figure 5E**) levels in the ovaries of CY/BUS-POF mice. Similarly, COS post-treatment increased MVH and Fragilis protein (**Figure 5D**) and mRNA (**Figure 5F**) levels in the ovaries of CY/BUS-POF mice.

**Figure 6:**
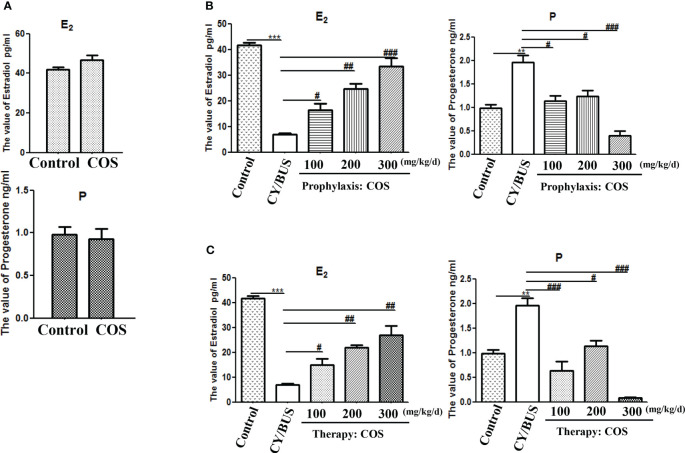
Serum levels of estradiol and progesterone. At endpoint after each treatment procedure, serum samples were obtained for the analysis. Concentrations of estradiol and progesterone were determined in duplicate using radioimmunoassay. **(A)** Serum hormone levels from mice with or without COS treatment. Top: estradiol, bottom: progesterone. **(B)** Serum levels of estradiol (Left) and progesterone (Right) were measured after prophylaxis procedure. **(C)** Serum levels of estradiol (Left) and progesterone (Right) from mice with therapy treatment procedure. n=6, Mean ± SEM. **p<0.01, ***p<0.001 vs Control group. #p<0.05, ##p<0.01, ###p<0.001 vs CY/BUS group.

Subsequently, the estradiol and progesterone in peripheral blood were measured (**Figure 6**). COS at a dose of 200 mg/kg/d did not change the levels of estradiol nor progesterone in peripheral blood of normal mice. CY/BUS-POF mice had significantly lower estradiol but higher progesterone than normal control mice (**Figure 6A**). Pre-treatment with different doses of COS inhibited the CY/BUS-induced estrogen level reduction the progesterone level increase (**Figure 6B**). COS post-treatment increased peripheral blood estradiol levels and decreased peripheral blood progesterone levels in CY/BUS-POF mice with a dose-dependent manner (**Figure 6C**).

### 3.3 COS regulated the levels of immune cytokines IL-2, TNF-α and IL-4 in the CY/BUS-POF mice ovaries

**Figure 7:**
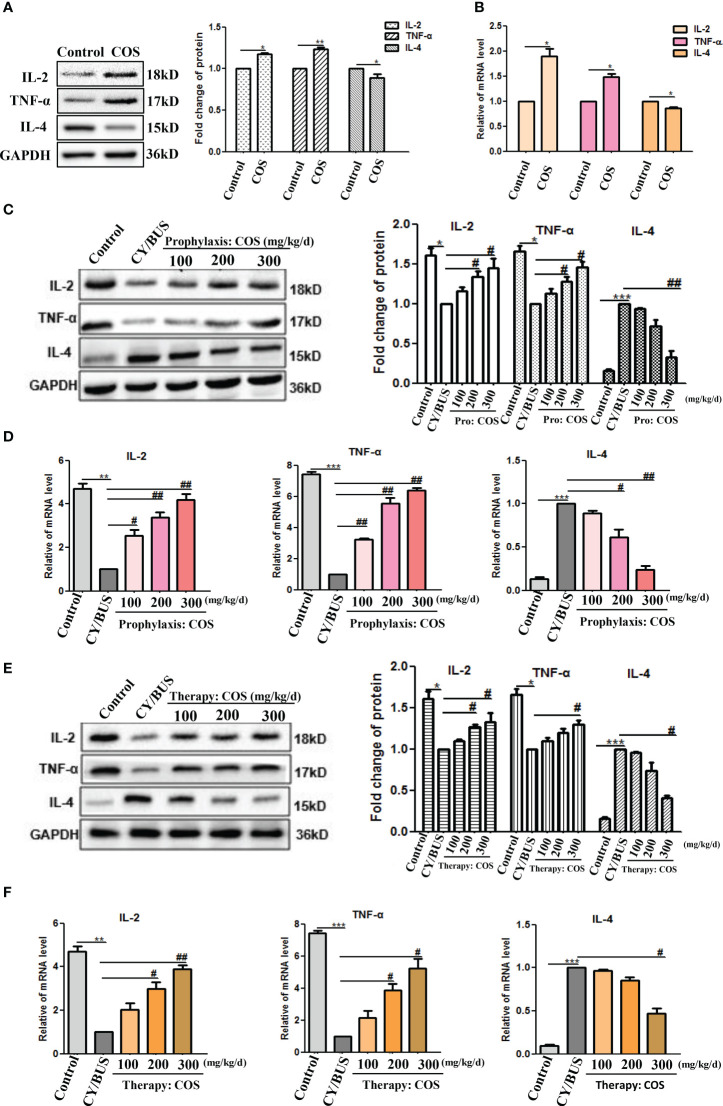
COS regulate the levels of immune cytokines IL-2, TNF-α and IL-4 in mice ovaries. At the endpoint of each treatment procedure, ovaries were collected then homogenized. Western blot performed on ovarian protein for IL-2, TNF-α and IL-4, qRT-PCR results showing mRNA expression of inflammation related genes IL-2, TNF-α and IL-4. A-B. IL-2, TNF-α and IL-4 protein **(A)** and mRNA **(B)** lever in ovaries from mice treated with or without COS (200 mg/kg. d). **(C)** IL-2, TNF-α and IL-4 proteins from mice after prophylaxis procedure were detected. Left: western blot protein bands. Right: western blot densitometry values analysis result. **(D)** IL-2, TNF-α and IL-4 mRNA detection results after prophylaxis procedure. **(E)** IL-2, TNF-α and IL-4 proteins results from mice after therapy procedure were detected. Left: western blot protein bands. Right: western blot densitometry values analysis result. **(F)** IL-2, TNF-α and IL-4 mRNA detection results after therapy procedure. n=3, Mean ± SEM. *p<0.05, **p<0.01, ***p<0.001 vs Control group. #p<0.05, ##p<0.01 vs CY/BUS group.

We employed Western blotting and qRT-PCR analyses to investigate the impact of COS on cytokine IL-2, TNF-α and IL-4 levels in the mice ovaries (**Figure 7**,**Supplementary Figure 2**). Results indicate that administration of COS at a dose of 200 mg/kg/d resulted in a slight upregulation of IL-2 and TNF-α expression and mRNA, while downregulating levels of IL-4 protein and mRNA (**Figures 7A, B**). In the prevention groups (**Figures 7C, D**), administration of COS was found to effectively prevent the decreased expression of protein and mRNA of IL-2 and TNF-α, as well as the increased expression of mRNA and protein of IL-4. Similarly, in the treatment groups (**Figures 7E, F**), COS was observed to effectively reverse the decreased expression of protein and mRNA of IL-2 and TNF-α, and the increased expression of mRNA and protein of IL-4.

### 3.4 COS protected CY/BUS-POF mice ovarian cells from heavy senescence

**Figure 8:**
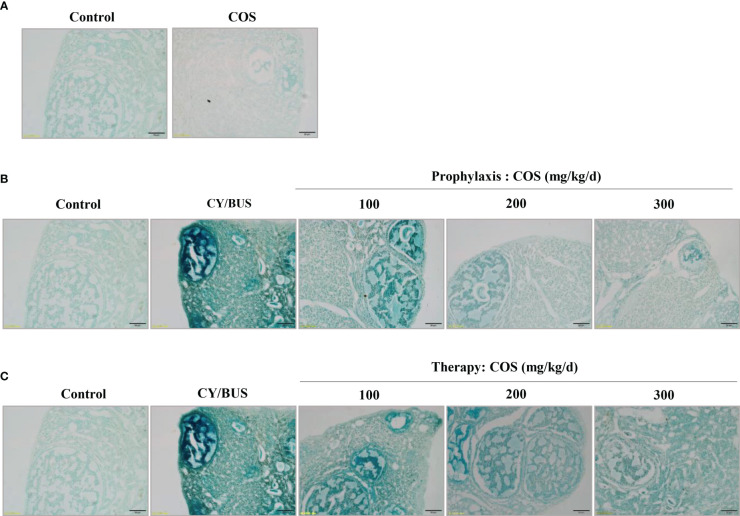
Senescence-associated β-galactosidase staining in mice ovarian tissue. The ovarian tissues were subjected to SA-β-Gal staining to assess the impact of COS and CY/BUS on the senescence of ovarian cells. **(A)** Ovarian section microphotographs after the SA-β-Gal staining patterns in mice that received either COS (200mg/kg/d) or no treatment. The staining intensity in the ovaries of the CY/BUS-induced POF mice was markedly higher compared to that of the control mice **(B, C)**. **(B)** Microphotographs depict the dose-dependent reduction in the SA-β-Gal staining intensity in the ovaries of mice that received COS prophylaxis. **(C)** Images demonstrate the dose-dependent decrease in the SA-β-Gal staining intensity in the ovaries of mice that received COS therapy. Scale bars = 50μm.

To investigate ovarian cells senescence, we performed SA-beta-gal staining. By observing the intensity of positive staining, it was found that the ovarian cells of COS-treated mice did not show strong senescence state compared with normal control mice (**Figure 8A**). The ovaries of CY/BUS-POF mice showed very marked cellular senescence. Compared with CY/BUS-POF mice, the positive level of SA-β-Gal staining in the ovaries of COS preventive treatment groups significantly decreased with dose-dependent manner (**Figure 8B**). Similar results were obtained in therapy treatment mice ovaries (**Figure 8C**).

**Figure 9:**
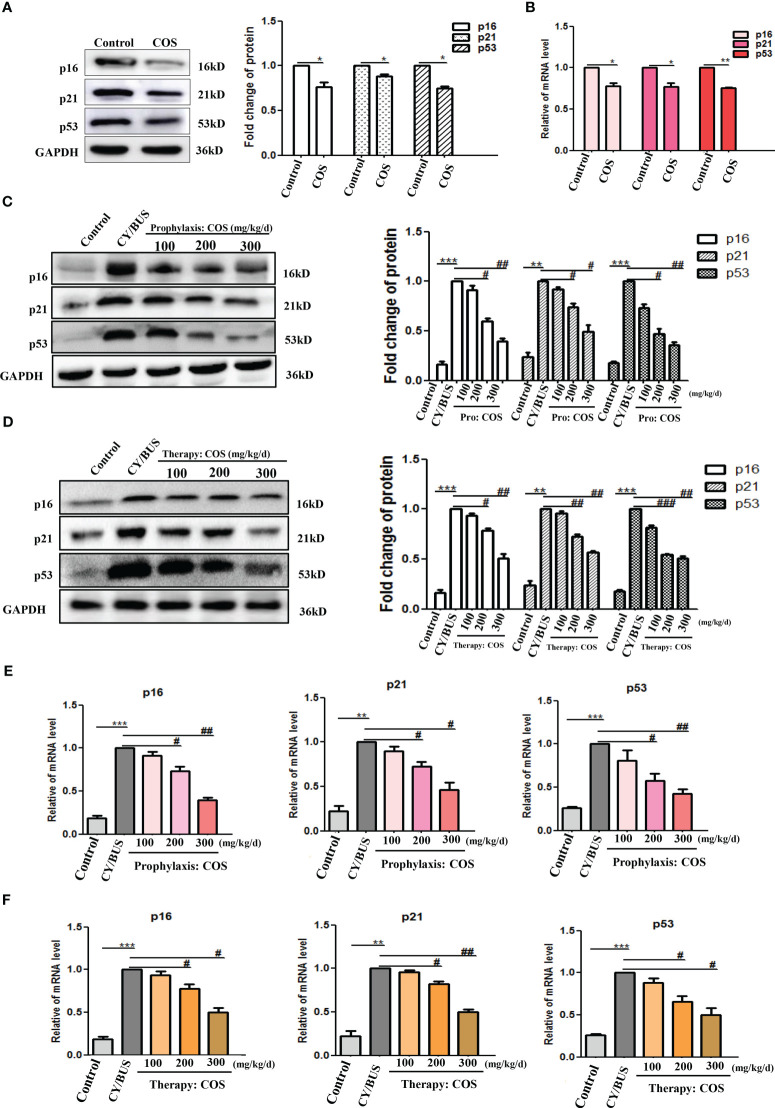
COS down-regulate p53/p21/p16 signaling axis in mice ovaries. After drug treatment, total protein and mRNA were extracted from mouse ovaries then analyzed by western blot and qRT-PCR respectively to determine the cellular senescence *via* p16, p21 and p53**. (A, B)** p16, p21 and p53 protein **(A)** and mRNA **(B)** lever in ovaries from mice treated with or without COS (200 mg/kg. d). **(C)** p16, p21 and p53 protein detection results from mice ovaries after prophylaxis procedure. Left: western blot protein bands. Right: western blot densitometry values analysis result. **(D)** p16, p21 and p53 protein detection results from mice ovaries after therapy procedure. Left: western blot protein bands. Right: western blot densitometry values analysis result. **(E, F)**. p16, p21 and p53 mRNA expression results in mice ovaries after prophylaxis procedure **(E)** and therapy procedure **(F)**. n=3, Mean ± SEM. *p<0.05, **p<0.01, ***p<0.001 vs Control group. #p<0.05, ##p<0.01 vs CY/BUS group.

The p53/p21/p16 signaling cascade is essential in the cellular response to DNA damage. Upregulation of p53/p21/p16 signaling has been observed in senescent germ cells. This study demonstrates that CY/BUS treatment activates the p53/p21/p16 senescence pathway in mouse ovaries, ultimately leading to germ cell senescence (**Figure 9**, **Supplementary Figure 3**). However, COS treatment can attenuate CY/BUS-induced senescence by downregulating p53/p21/p16 signaling. Specifically, the pretreatment groups exhibited a dose-dependent decrease in p53, p21, and p16 levels in the ovaries (**Figures 9C, E**), and the posttreatment group demonstrated a significant reduction in these signaling molecules as well (**Figures 9D, F**).

## 4 Discussion

POF remains an enigma and a challenge for physicians due to the lack of effective treatment available currently (2). We previously reported that COS has a protective effect on ovarian damage *in vitro* and *in vivo* (28, 31). However, the exact mechanism by which COS prevents ovarian damage still needs to be fully explored. In this study, we explored the regulatory effect of COS on the systemic immune status of POF mice, as well as its protective effects on ovarian reproductive function, immune microenvironment, and aging status. We found that COS helps to maintains the function thymus (one of the central immune organs) and spleen (one of the peripheral immune organs), enhances macrophage phagocytic activity, improves the systemic immunity of POF mice, and regulates the local IL-2、IL-4 and TNF-α immune balance and the p53/p21/p16 signaling axis to improve the immune microenvironment of the ovaries. Collectively, our data support the notion that COS may serve as a pivotal immunomodulator and a prophylactic and therapeutic agent for POF caused by chemicals such as CY/BUS.

The thymus serves as a crucial immunological organ wherein T cells undergo development and maturation. Its stromal cells, including thymic epithelial cells, are capable of producing various cytokines, including IL-2, IL-4, and TNF-α, that contribute to the regulation of cell-mediated immunity (32). Structurally, the thymus is composed of two regions: the cortex and medulla. The cortex primarily consists of immature T cells or thymocytes, as well as some macrophages. The cortical epithelial cells produce hormones and cytokines that facilitate the differentiation and development of thymocytes (33). The medulla, on the other hand, contains multiple types of epithelial cells, such as epithelial reticular cells, mature T cells, macrophages, and thymic corpuscles. The spleen, as the principal peripheral immune organ harboring mature T and B cells, is the largest immune organ in the human body. It comprises the white pulp, red pulp, and marginal zone. The white pulp includes the peripheral lymphatic sheath and lymphoid nodule, whereas the red pulp is constituted by the splenic cord and splenic sinus (34). Additionally, peritoneal resident macrophages play a crucial role in the immune system as the first line of defense against invading pathogens (35). They are involved in a variety of immune functions, including phagocytosis, antigen presentation, and cytokine production (36). The immune status of an individual is closely related to the function and activity of peritoneal macrophages. Some studies have shown that the number and function of peritoneal macrophages can be altered in various disease states, such as infections, cancer, and autoimmune disorders (36–38). In this study, we have demonstrated the efficacy of COS in mitigating the deleterious effects of CY/BUS-induced damage on the thymus and spleen in both prophylaxis and therapy groups. Since abdominal macrophages are innate immune cells, their phagocytic ability can serve as a reliable indicator of non-specific immunity (39). Results from the neutral red phagocytosis experiment revealed that the phagocytic ability of abdominal macrophages was stronger in both the COS prevention and treatment groups compared to the CY/BUS group. This suggests that COS can activate the non-specific immune function of mice.

Ovarian senescence refers to a reduction in the number of follicles, resulting in changes in follicle morphology and premature follicle atresia in clinical settings (40). The secretion of estrogen and progesterone declines as the follicle pool becomes exhausted (41). During gestation, the fetal oogonium undergoes mitosis, with the number of germ cells reaching 600-700 million by the 20th week of gestation. Between the 8th and 13th weeks, two-thirds of these cells enter the mitotic phase and become primary oocytes, with mitosis completing by the 28th week of gestation (42). At birth, the number of germ cells ranges from 100 to 200 million, decreasing to approximately 500,000 at menarche. The number of oocytes continues to decline during the reproductive period, with fewer than 100 remaining at menopause, when they lose their response to gonadotrophin (43). While several opinions exist on the reasons for ovarian senescence, most scholars believe that the exhaustion of the follicular pool is the major cause (44, 45). Our study evaluated the function of the mice ovary according to the ovary index, the structure of the ovary, the expression level of ovarian germ cell marker MVH/Fragilis and the peripheral blood level of estrogen and progesterone. The results demonstrated that different doses of COS can improve follicular development and hormone secretion in POF mice, regardless of whether administered prophylactically or therapeutically. These findings suggest that COS is an effective treatment for CY/BUS-induced POF in mice.

Immunity is closely related to the occurrence and development of POF (46). As a putative immunomodulator, COS has been reported in previous articles to protect mouse follicular granulosa cells by inhibiting peroxidation stress and damaging related pathways like HIF-1α signaling (31) or SESN2/NRF2 signaling (28). Our previous study shows that COS can improve the inflammation and oxidative stress of ovarian granulosa cells in PCOS patients (26). Here, we focus on Th1/Th2 balance in the ovarian immune microenvironment. The subpopulation of T-lymphocytes, known as helper T-cells is crucial for immune function in the human body. Empirical evidence has established that under normal physiological conditions, the Th1 and Th2 subsets maintain a functional balance (47). However, when cell-mediated immunity is triggered, Th1 predominance occurs, whereas Th2 predominance occurs when humoral immunity is activated. Recent researches have demonstrated that polarization between Th1 and Th2 is more complex and can be modulated by altering cellular conditions such as abundance of intracellular glutathione, nutrients, and hormones (48, 49). The cytokines IL-2 and TNF-α are secreted by Th1 cells, whereas IL-4 is the most specific cytokine secreted by Th2 cells (50). The results of the cytokine expression analysis in the ovary indicate that the administration of COS resulted in an increase in the expression levels of IL-2 and TNF-α in all COS treatment groups compared to the CY/BUS group. Conversely, the expression level of IL-4 decreased in a dose-dependent manner. These findings suggest that COS may promote the proliferation of T cells to a certain extent by regulating Th1/Th2 and maintaining a balance between humoral and cell-mediated immunity in mice ovaries.

The signaling pathway involving p53/p21/p16 is crucial for the cellular reaction to DNA damage (51). Activation of p53/p21/p16 signaling has been detected in senescent germ cells (52). We evaluated the condition of ovarian recession according to the level of ovarian senescence genes p53/p21/p16. The results showed that COS pretreatment and post-treatment inhibited p53/p21/p16 signaling, and improved the ovarian cell senescence presented by SA-β-galactosidase staining. The outcomes demonstrated the beneficial impact of COS on ovarian cell senescence induced by CY/BUS.

For women, effective prevention of POF to mitigate the damage and symptoms caused by diminished ovarian function imply significant physiological and psychological implications (53). Our experimental findings on COS-administered normal mice indicate that despite the imperceptible changes in thymus and spleen structure, continuous COS gavage led to an increase in the ratio of immune organs to body weight. As well as, COS promoted the phagocytosis activity of peritoneal macrophages. Our analysis of ovarian function showed that COS had no effect on the ovarian index of normal mice, but significantly enhanced the quality of ovarian germ cells, as evidenced by elevated levels of germ cell markers MVH and Fragilis protein and mRNA. Further analysis of the normal ovaries of COS-treated mice revealed a regulation of immune factors IL-2, TNF-α and IL-4, and a down-regulation of cell aging signal p16/p21/p53. Combining the results from the prevention groups, we conclude that the preventive mechanism of COS against CY/BUS lies in its ability to strongly resist immune and reproductive from damage by optimizing the system and ovarian local immune of mice. These findings provide experimental support for considering COS as a health care prophylactic drug for reproductive women in the future.

## 5 Conclusions

In summary, our investigation has demonstrated that COS not only enhances the immune functions in the ovary to facilitate the development of ovarian germ cells but also exhibits immunoregulatory effects on immune system, especially on innate immune cells. Additionally, COS regulates the equilibrium between local cellular immunity and humoral immunity in the ovary, while also modulating the p53/p21/16 signaling pathway to prevent ovarian injury and senescence in CY/BUS-induced POF mice. Our findings demonstrate that COS is a potential agent against POF.

## Data availability statement

The original contributions presented in the study are included in the article/**Supplementary Material**. Further inquiries can be directed to the corresponding authors.

## Ethics statement

The animal study was reviewed and approved by The Experimental Animal Welfare and Ethics Committee of Nanchang University.

## Author contributions

YZ and XL contributed to the conception and design of this work. XL, HY, TS, CH, YH and XF conducted the experiments. XL analyzed the data. XL and ZZ wrote the manuscript, YZ and XD reviewed edited the manuscript. All authors contributed to the article and approved the submitted version.
