# Supplementary material for: Immunity and reproduction protective effects of Chitosan Oligosaccharides in Cyclophosphamide/Busulfan-induced premature ovarian failure model mice
Source: Front Immunol. 2023 May 9;14:1185921. doi: 10.3389/fimmu.2023.1185921 (PMC10203494; doi:10.3389/fimmu.2023.1185921)
Supplement: Supplementary file 1 [file DataSheet_1.docx]

*For Frontiers in Immunology*

**Supplemental information**

# Immunity and Reproduction Protective Effects of Chitosan Oligosaccharides in Cyclophosphamide/Busulfan-induced Premature Ovarian Failure Model Mice

Xiaoyan Li, Haifeng Ye, Tie Su, Chuan Hu, Yaoqi Huang, Xinxin Fu, Zhisheng Zhong *, Xuelian Du *, Yuehui Zheng *

**Supplementary Table 1. Primers used for quantitative-PCR**

| **Gene** | **Forward primer (5'-3')** | **Reverse primer (5'-3')** |
| --- | --- | --- |
| GAPDH  MVH  Fragilis  IL-2  IL-4  TNF-α  p16  p21  p53 | CAGGTGGTCTCCTCGACTT  GTGTATTATTGTAGCACCAACTCG  CTGGTCCCTGTTCAATACACTCTT  TTTTACTTGCCCAAGCAGGC  CCATATCCACGGATGCGACA  AGGCACTCCCCCAAAAGATG  TCTTTGTGTACCGCTGGGAAC  GTCGCTGTCTTGCACTCTGG  GTCGGGAAGTGACCACCAAT | CCAAATTCGTTGTCATACCA  CACCCTTGTACTATCTGTCGAACT  CAGTCACATCACCCACCATCTT  AAAGTCCACCACAGTTGCTGA  AAGCACCTTGGAAGCCCTAC  CCACTTGGTGGTTTGTGAGTG  GCATCGCTAGAAGTGAAGCTAAGAA  CCAATCTGCGCTTGGAGTGATA  ATGTCCCACACGGAGACAAC |

**Supplementary Table 2. Effect of COS on ovarian index in normal mice**

| **Group** | **Weight  (g)** |  |  | **Ovary** | |  |
| --- | --- | --- | --- | --- | --- | --- |
|  |  |  |  | **Weight  (g)** | **Index (‰)** |  |
|  |  |  |  |  |  |  |
| **Control** | **34.16± 2.035** |  |  | **0.0212±0.0032** | **0.620±0.084** |  |
| **200COS** | **36.67±2.183** |  |  | **0.0229±0.0023** | **0.626±0.054** |  |

**Supplementary Table 3. Changes in ovarian index in COS prevention groups**

| **Group** | **Weight  (g)** |  |  | **Ovary** | |  |
| --- | --- | --- | --- | --- | --- | --- |
|  |  |  |  | **Weight  (g)** | **Index (‰)** |  |
|  |  |  |  |  |  |  |
| **Control** | **34.16± 2.035** |  |  | **0.0212±0.0032** | **0.620±0.084** |  |
| **CY/BUS** | **29.86±3.476** |  |  | **0.0126±0.0022** | **0.424±0.075***** |  |
| **Prevention-100** | **31.82±0.860** |  |  | **0.0155±0.0048** | **0.489±0.157** |  |
| **Prevention-200** | **32.93±1.536** |  |  | **0.0168±0.0020** | **0.511±0.053###** |  |
| **Prevention-300** | **33.98±1.166** |  |  | **0.0192±0.0027** | **0.565±0.084###** |  |

***p<0.001, vs Control group. ###p<0.001, vs CY/BUS group.

**Supplementary Table 4. Changes in ovarian index in COS treatment groups**

| **Group** | **Weight  (g)** |  |  | **Ovary** | |  |
| --- | --- | --- | --- | --- | --- | --- |
|  |  |  |  | **Weight  (g)** | **Index (‰)** |  |
|  |  |  |  |  |  |  |
| **Control** | **34.16± 2.035** |  |  | **0.0212±0.0032** | **0.620±0.084** |  |
| **CY/BUS** | **29.86±3.476** |  |  | **0.0126±0.0022** | **0.424±0.075***** |  |
| **Treatment-100** | **30.81±0.705** |  |  | **0.0142±0.0035** | **0.461±0.110** |  |
| **Treatment-200** | **31.71±1.080** |  |  | **0.0151±0.0029** | **0.477±0.097#** |  |
| **Treatment-300** | **32.74±1.076** |  |  | **0.0173±0.0096** | **0.525±0.281#** |  |

***p<0.001, vs Control group. #p<0.05, vs CY/BUS group.


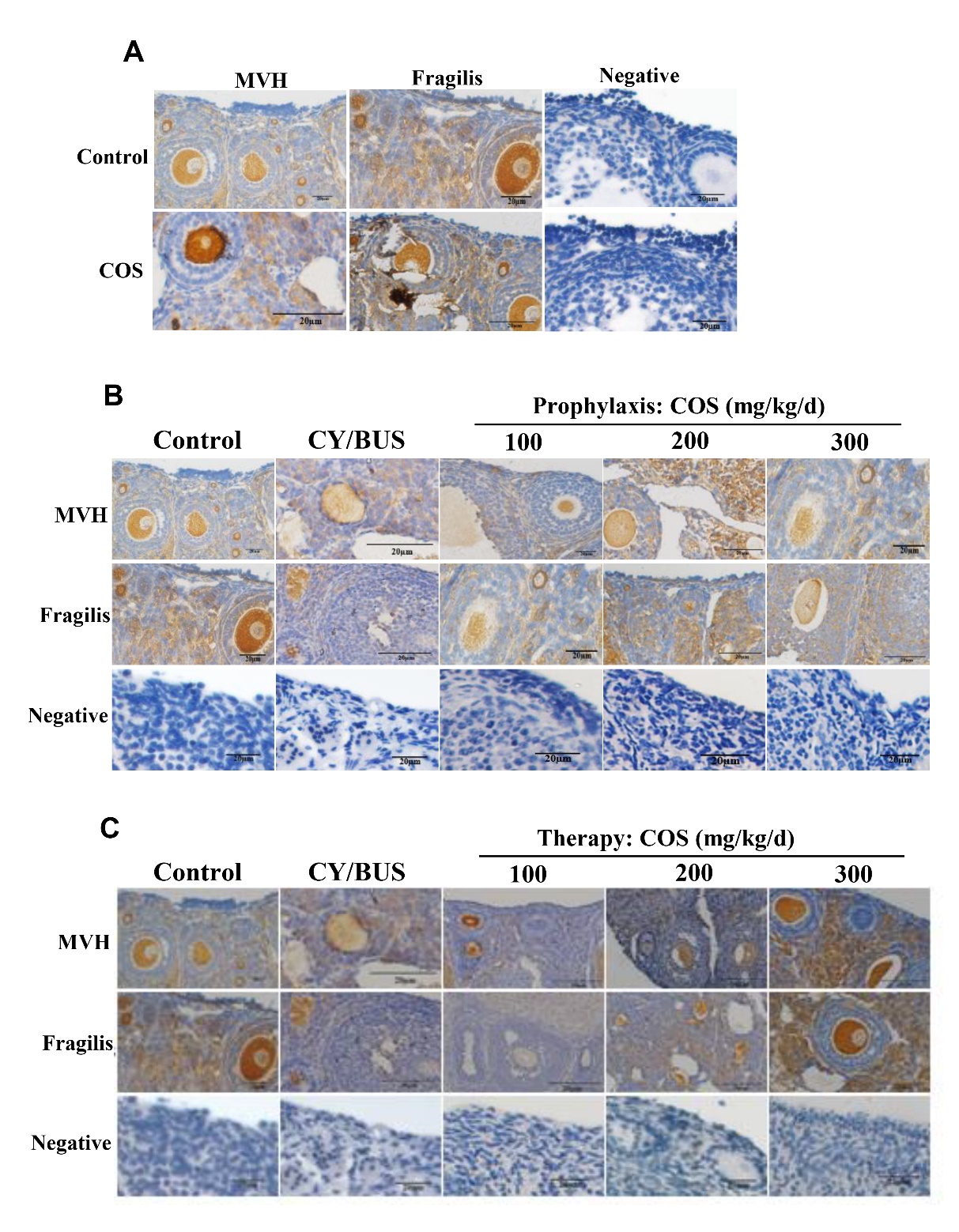


**Supplementary Figure 1. Immunohistochemistry (IHC) essay for MVH and Fragilis in paraffin-embedded ovarian tissues.** After drug treatment, the ovaries were fixed and paraffin-embedded, then sliced for immunohistochemical staining in maximum cross section. **A:** Staining of MVH and Fragilis in single COS or none treated mice ovaries. **B:** Immunohistochemistry for MVH and Fragilis levels in mice ovaries treated with prophylaxis procedure. **C:** Immunohistochemical ovaries images for MVH and Fragilis from mice treated with therapy procedure. MVH and Fragilis were strongly positive in ovarian germ cells, showing as dark brown.


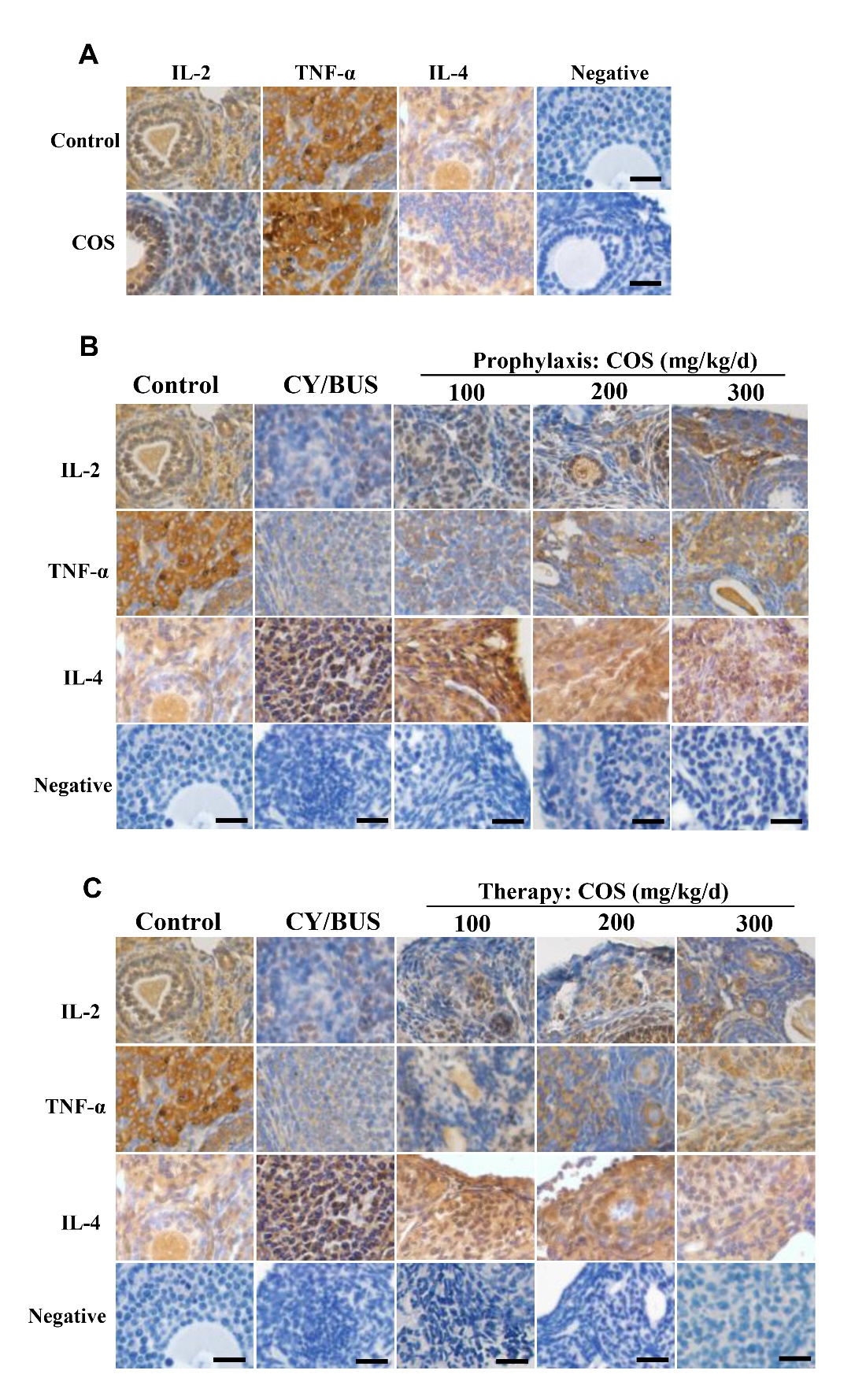


**Supplementary Figure 2. Immunohistochemistry essay for IL-2, TNF-α and IL-4 in paraffin-embedded ovarian tissues.** After the experimental treatment, the ovaries were collected, fixed, paraffin-embedded and sectioned, then photographed after immunohistochemical staining in maximum cross section. **A:** IL-2, TNF-α and IL-4 expression results in ovaries from mice after COS (200 mg/kg. d) treatment or none. **B**: IL-2, TNF-α and IL-4 staining results after prophylaxis treatment procedure. **C:** IL-2, TNF-α and IL-4 staining results after therapy treatment procedure.


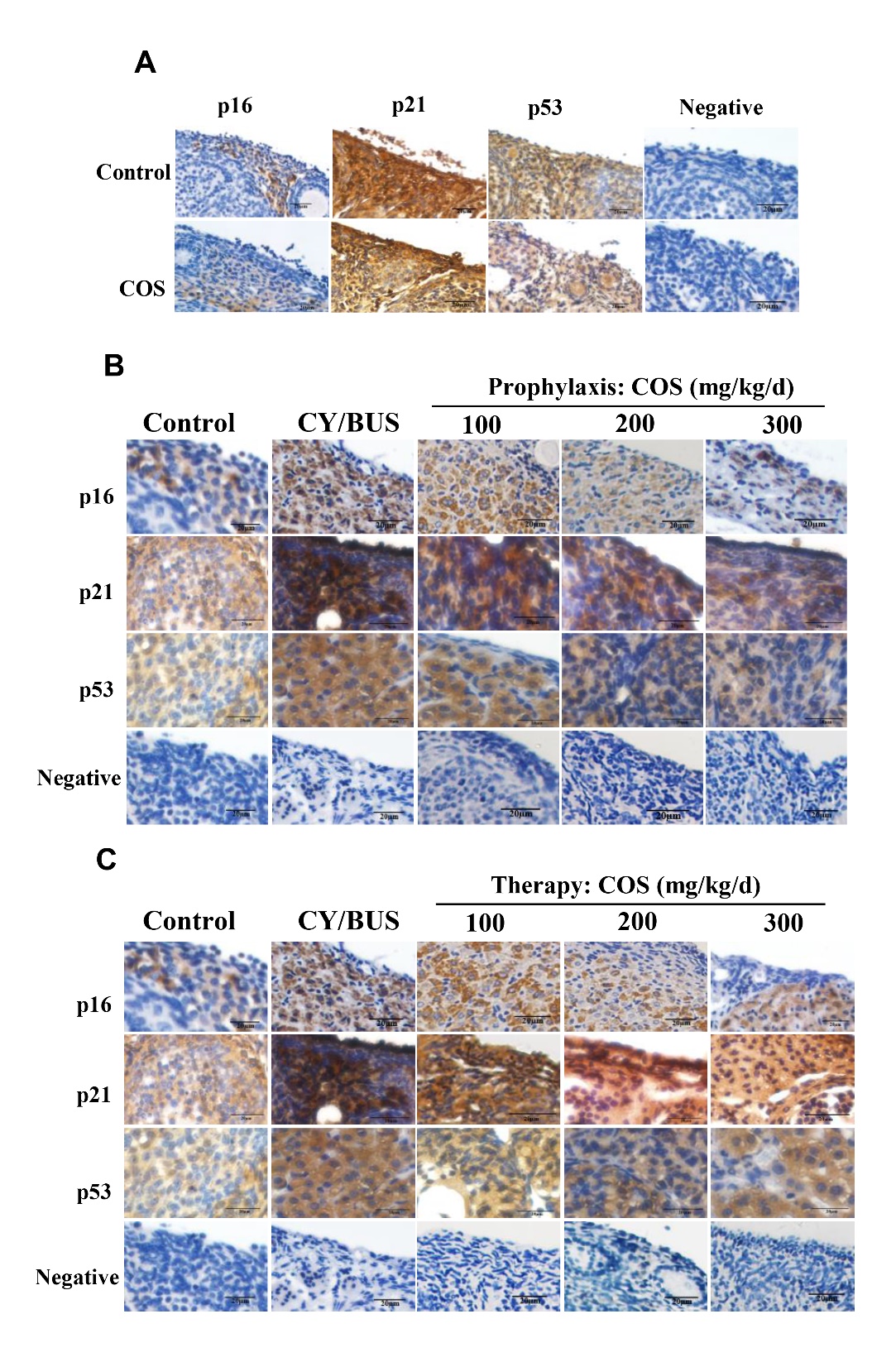


**Supplementary Figure 3. Immunohistochemical (IHC) staining of ovarian tissues with p16, p21 and p53 antibodies.** At each treatment endpoint, the ovaries were fixed and paraffin-embedded, then sliced for immunohistochemical staining in maximum cross section. **A:** Staining of p16, p21 and p53 in single COS or none treated mice ovaries. **B:** Immunohistochemistry for p16, p21 and p53 levels in mice ovaries treated with prophylaxis procedure**. C:** Immunohistochemical ovaries images for p16, p21 and p53 from mice treated with therapy procedure**.**
